# Post-training activation of Rac1 in the basolateral amygdala is required for the formation of both short-term and long-term auditory fear memory

**DOI:** 10.3389/fnmol.2015.00065

**Published:** 2015-11-04

**Authors:** Qinqin Gao, Wenqing Yao, Junjun Wang, Tong Yang, Cao Liu, Yezheng Tao, Yuejun Chen, Xing Liu, Lan Ma

**Affiliations:** State Key Laboratory of Medical Neurobiology, Collaborative Innovation Center for Brain Science, School of Basic Medical Sciences and the Institutes of Brain Science, Fudan UniversityShanghai, China

**Keywords:** Rac1, fear conditioning, BLA, memory formation, STM, LTM

## Abstract

Rac1, a member of the Rho family of small GTPases, is crucial for morphological changes of the mature neuronal synapse including spine formation and activity-dependent spine enlargement, while its role in the formation of associated memories, such as conditioned fear memory, is not clear. Here, we report that selective deletion of Rac1 in excitatory neurons, but not in parvalbumin inhibitory neurons, impaired short- and long-term memories (STM and LTM) of fear conditioning. Conditional knockout of Rac1 before associative fear training in the basolateral amygdala (BLA), a key area for fear memory acquisition and storage, impaired fear memory. The expression of dominant-negative mutant of Rac1, or infusion of Rac1 inhibitor NSC23766 into BLA blocked both STM and LTM of fear conditioning. Furthermore, selective inhibition of Rac1 activation in BLA immediately following fear conditioning impaired STM and LTM, demonstrating that fear conditioning-induced Rac1 activation in BLA plays a critical role in the formation of both STM and LTM of conditioned fear.

## Introduction

Fear conditioning is a classic model of associative fear learning, which is critical for animals to trigger adaptive behaviors in response to dangerous environmental threats. In a typical fear conditioning protocol, the animals are exposed to a neutral conditioned stimulus (CS), such as tone or the context of the conditioning chamber, paired with an aversive unconditioned stimulus (US), such as an electric footshock. After this CS-US association learning, the CS could elicit a set of defensive responses, such as freezing (Kim and Jung, 2006).

Studies have shown fear conditioning involves integration of CS and US inputs in the lateral amygdala (LA) and fear memory is stored in the basal amygdala (BA; Long et al., 2013) . The alterations in signaling and synaptic plasticity in the basolateral amygdala (BLA, composed of LA and BA) encode key aspects of the fear learning (Schafe et al., 2001; Long et al., 2013; Xu and Wang, 2013). Fear conditioning involves different stages of memory. Initial learning of the CS-US association during fear training is referred to as fear memory acquisition. During the 24-h period following fear learning, short-term memory (STM) which lasts minutes to hours consolidates into long term memory (LTM), which lasts days, weeks, or even a lifetime (McGaugh, 1966). Lesions and functional inactivation of BLA interfere with fear conditioning (Phillips and LeDoux, 1992; Baldi and Bucherelli, 2015), and fear conditioning induces neural plasticity in BLA (Quirk et al., 1995; Rogan et al., 1997; Goosens and Maren, 2004; Schafe et al., 2005). Furthermore, recent studies have indicated that activation of a number of membrane-bound receptors and intracellular signaling molecules in BLA is required for fear conditioning. NMDA receptors (NMDARs) and metabotropic glutamate receptors (mGluRs) are proved to contribute to the formation of STM and LTM. Blockade of the NR2B subunit of the NMDAR in the amygdala before fear conditioning impairs the formation of STM and LTM (Rodrigues et al., 2001). Infusion of mGluR5 antagonist into the amygdala prior to fear conditioning impairs both STM and LTM (Rodrigues et al., 2002). Multiple signal cascades including cAMP/PKA and ERK/MAPK have been implicated in memory consolidation. Fear conditioning results in a transient activation of ERK/MAPK in the amygdala and perfusion of PKA or ERK inhibitors into LA before fear conditioning causes the impairment of LTM (Schafe and LeDoux, 2000; Schafe et al., 2000; Moita et al., 2002). Evidence indicates that the molecules participating in neuronal morphology changes are needed for fear memory formation as well. Infusion of actin cytoskeleton assembly inhibitors into the amygdala immediately before fear conditioning interferes with the formation of LTM (Fischer et al., 2004; Mantzur et al., 2009).

Ras-related C3 botulinum toxin substrate 1 (Rac1) is a member of the small Rho GTPase family and a master protein of cytoskeletal changes (Jaffe and Hall, 2005; Newey et al., 2005). Like all GTP-binding proteins, Rac1 cycles between an inactive GDP-bound state and an active GTP-bound state. Rac1 is activated by guanine nucleotide exchange factors (GEFs), which catalyzing the exchange of GDP-bound inactive forms for GTP-bound active forms. The active Rac1 activates downstream signaling pathways to control a wide range of the functions of neurons, including cell migration, neuronal morphogenesis, and synapse formation (Luo, 2000; Tashiro et al., 2000; Etienne-Manneville and Hall, 2002; Heasman and Ridley, 2008). The involvement of Rac1 in fear memory has been also reported. Rac translocation and activation are increased in the hippocampus following associative fear conditioning in mice, and these increases can be blocked by infusion of the NMDA receptor channel blocker before fear conditioning (Martinez et al., 2007). The activation of cerebral Rac1 before training enhances fear memory (Diana et al., 2007), and extinction of contextual fear memory requires down regulation of Rac1 (Sananbenesi et al., 2007). Rac also regulates forgetting in Drosophila and rats, and the activation of Rac enhances, while inhibition of Rac impairs forgetting (Shuai et al., 2010; Jiang et al., 2015). Injection of Rac1 inhibitor NSC23766 into BLA immediately after memory retrieval disrupted the reconsolidation of auditory Pavlovian fear memory (Wu et al., 2014). However its role in fear memory acquisition and consolidation is not clear.

## Material and Methods

### Subjects

Rac1^flox/flox^, CaMKIIα-cre and PV-cre mice were obtained from Jackson Laboratories (Stock number: 005550, 005359, 012358). Rac1^flox/flox^ mice were crossed with CaMKIIα-cre or PV-cre mice to generate conditional Rac1 knockout mice with Rac1 ablated primarily in glutamatergic excitatory neurons or PV interneurons. Seven-week-old male C57BL/6 mice were purchased from the Shanghai SLAC Laboratory Animal Co., Ltd. 8–10 weeks old mice were used for behavioral experiments. All subjects were maintained on a reversed 12 h light/dark cycle at 25°C with food and water available ad libitum. All animal treatments were strictly in accordance with the National Institutes of Health Guide for the Care and Use of Laboratory Animals and were approved by Animal Care and Use Committee of Shanghai Medical College of Fudan University.

### Virus Construct and Packaging

Plasmids encoding Rac1 dominant negative mutant (Rac1-DN) were provided by Dr. Xiaobing Yuan (Institutes of Neuroscience, Shanghai Institutes for Biological Science, Chinese Academy of Science, China). A synapsin promoter-driven AAV vector was used to express Rac1-DN and EGFP reporter by cloning Rac1-DN-IRES-EGFP into the AAV BamHI/EcoRI restriction site. AAV2/8 with a titer exceeding 1 × 10^12^ vg/mediolateral (ML) was used for injection (Neuron Biotech Co., Ltd.). To obtain mice with Rac1 conditionally knocked out in BLA, the AAV encoding Cre recombinase and EGFP reporter (AAV-EF1α-EGFP-T2A-Cre) or control virus (AAV-EF1α-EGFP-T2A) produced by Neuron Biotech Co., Ltd. was injected into BLA of Rac1^flox/flox^ mice.

### Stereotactic Surgery

Mice were anaesthetized (chloral hydrate: 400 mg/kg, i.p.) and implanted with 26 gauge pedestal guide cannula (Plastics One, Inc.) targeting to BLA according to the following coordinates: anterioposterior (AP): −1.7 mm; ML: ± 3.2 mm; dorsoventral (DV): –3.5 mm. The mice were fixed with dental acrylic cement. Dummy cannulas were inserted into the guide cannulas following surgery and remained in place until the time of drug injection. The intended stereotaxic coordinates for AAV injection were: AP: −1.7 mm; ML: ± 3.2 mm; DV: –4.5 mm. The virus was injected 0.5 μl each side at a rate of 0.1 μl/min. After injection, the needle (33 Gauge, Hamilton Robotics, Inc.) stayed for five additional minutes before it was slowly withdrawn.

### Drugs and Intracranial Injections

NSC23766 (Tocris) was dissolved in saline (10 μg/μl, 0.5 μl/side). The control group was treated with saline (0.5 μl/side). The drug was freshly prepared for the experiments. To inject NSC23766 or saline into BLA, the 33 Gauge mating internal cannulas with 1 mm projection (Plastics One, Inc.) were used by connecting with an infusion pump (BAS) and the liquid was injected at a rate of 0.1 μl/min. After injection, the internal cannula stayed for two additional minutes before withdrawal.

### Cued Fear Conditioning

Mice were handled once every day for 3 days. A computer-controlled fear conditioning system (MED Associates) was used. The conditioning chamber (30 × 25 × 33 cm^3^) was composed of stainless steel rod floor surrounded by a sound-attenuating box. During the memory acquisition phase (8 min), the mice received three training trials with a 2-min intertrial interval. In each trial, the mice were exposed to a tone (CS: 2800 Hz, 80 dB, 30 s) that co-terminated with a footshock (US: 0.7 mA, 0.5 s). Then the mice were re-exposed to the conditioning chamber with a novel environment 1 h or 24 h after training for 3-min adaptation followed by 3-min tone. The freezing percentage during each CS was automatically analyzed to reflect the memory acquisition, STM and LTM of fear conditioning.

### Immunohistochemistry

The animals were anesthetized by chloral hydrate (400 mg/kg, i.p.) and transcardially perfused with saline, followed by 4% paraformaldehyde in PBS. Then the brains were quickly removed and post fixed with 4% paraformaldehyde at 4°C for about 4 h and stored in 30% PBS-buffered sucrose solution for 72 h. Coronal sections (30 μm) were cut with a cryostat (Leica) and washed in PBS, blocked with blocking buffer (10% donkey serum in PBS containing 0.3% Triton X-100) for 1 h, and incubated in mouse anti-Rac1 antibody (1:20, Cytoskeleton) and rabbit anti-PV antibody (1:1000, Swant) overnight at 4°C. Sections were subsequently rinsed with PBS, incubated with secondary antibody (1:50,000, Jackson ImmunoResearch) for 2 h at room temperature, and rinsed with PBS. As the last step, the slices were mounted with anti-quenching mounting medium (Thermo Fisher Scientific) and then the coverslips were applied. The images were captured under a LSM 510 laser confocal fluorescence microscope (Carl Zeiss). Raw images obtained from three non-contiguous sections of BLA were used for determination of the integrated optical density (IOD) of the stained structures to quantify Rac1 expression in GFP positive neurons.

### Statistical Analysis

The data are presented as mean ± SEM, and were statistically analyzed using Sigmaplot 12.5. For the open field task, data were analyzed by two-way analysis of variance (ANOVA) and *t*-test. For the fear conditioning task, data were analyzed by two-way ANOVA followed by Bonferroni’s *post hoc* test with sessions as a within-subjects factor and genotype or drug treatment as a between-subjects factor. Values of *p* < 0.05 are defined as statistically significant.

## Results

### Rac1 in Excitatory Neurons is Required for STM and LTM of Conditioned Fear

**Figure 1:**
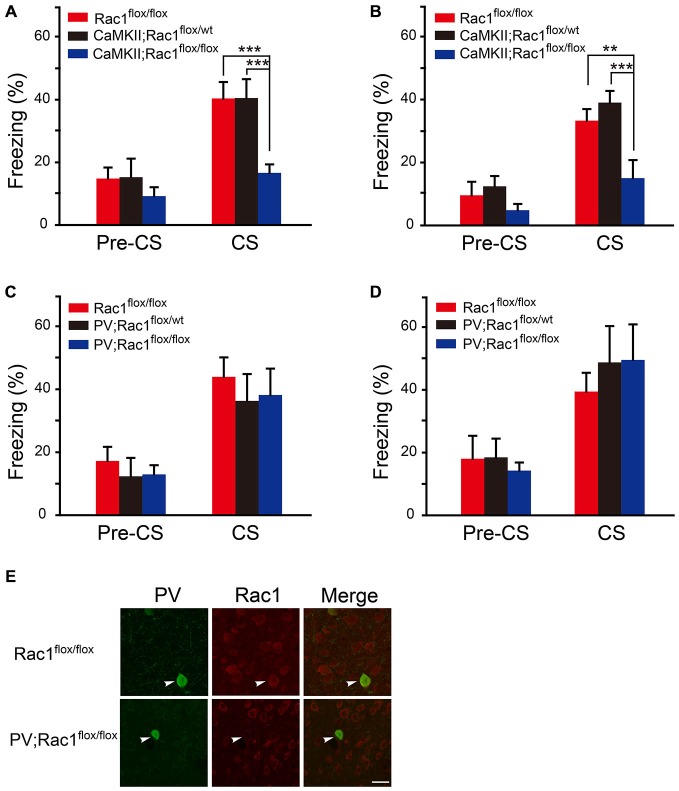
**Conditional knockout of Rac1 in CaMKIIα-positive excitatory neurons impaired short-term and long-term fear memory. (A,B)** Ablation of Rac1 in the CaMKII excitatory neurons decreased freezing at 1 h (**A**, Rac1^flox/flox^: *n* = 6, CaMKII; Rac1^flox/wt^: *n* = 6, CaMKII; Rac1^flox/flox^: *n* = 9, ***p < 0.001 vs. Rac1^flox/flox^ and CaMKII; Rac1^flox/wt^ group) and 24 h (**B**, Rac1^flox/flox^: *n* = 6, CaMKII; Rac1^flox/wt^: *n* = 8, CaMKII; Rac1^flox/flox^: *n* = 8, **p < 0.01 vs. Rac1^flox/flox^ group, ***p < 0.001 vs. CaMKII; Rac1^flox/wt^ group) auditory fear memory tests. **(C,D)** Ablation of Rac1 in the PV inhibitory neurons did not change freezing at 1 h (**C**, Rac1^flox/flox^: *n* = 6, PV; Rac1^flox/wt^: *n* = 8, PV; Rac1^flox/flox^: *n* = 8) or 24 h (**D**, Rac1^flox/flox^: *n* = 6, PV; Rac1^flox/wt^: *n* = 6, PV;Rac1^flox/flox^: *n* = 7) auditory fear memory test. **(E)** Immunofluorescence staining of Rac1 (red) and PV (green) in basolateral amygdala (BLA) of PV; Rac1^flox/flox^ and Rac1^flox/flox^ mice. Scale bar: 25 μm.

The role of Rac1 in fear memory was tested in Rac1 conditional knockout mice which lack Rac1 in excitatory (CaMKII; Rac1^flox/flox^) or inhibitory (PV; Rac1^flox/flox^) neurons. During the training session, the mice were exposed to three trials of 30 s tone co-terminated with one footshock. The response of Rac1 conditional knockout mice to footshock was similar to that of the wild type (Rac1^flox/flox^) mice, with increasing freezing levels from the 1st to 3rd trial (data not shown). After three CS-US parings of fear training, mice were exposed to a novel context and presented with the same CS (tone) 1 h or 24 h after training to test STM and LTM. As shown in Figures 1A,B when presented with CS 1 h and 24 h after training, the mice with Rac1 deletion in CaMKIIα-positive neurons (CaMKII;Rac1^flox/flox^) displayed significantly decreased freezing levels compared with their wild-type (Rac1^flox/flox^) and heterozygous (CaMKII;Rac1^flox/wt^) littermates (Figure 1A, 1 h, two-way ANOVA, *F*_genotype×session(2,36)_ = 3.390, *p* = 0.045; Figure 1B, 24 h, two-way ANOVA, *F*_genotype×session(2,38)_ = 6.393, *p* = 0.004), suggesting Rac1 deletion in excitatory neurons impairs both short-term and long-term fear memory formation. In contrast, as shown in Figures 1C–E, the mice with Rac1 deletion in PV inhibitory neurons (PV; Rac1^flox/flox^) showed no change in freezing level compared with their wild-type (Rac1^flox/flox^) and heterozygous (PV; Rac1^flox/wt^) littermates, when presented with CS 1 h or 24 h after training (Figure 1C, 1 h, two-way ANOVA, *F*_genotype×session(2,30)_ = 0.0506, *p* = 0.951; Figure 1D, 24 h, two-way ANOVA, *F*_genotype×session(2,32)_ = 0.23, *p* = 0.796). These results suggest that Rac1 in excitatory neurons is required for STM and LTM of conditioned fear.

### Rac1 in BLA is Required for STM and LTM of Fear Conditioning

**Figure 2:**
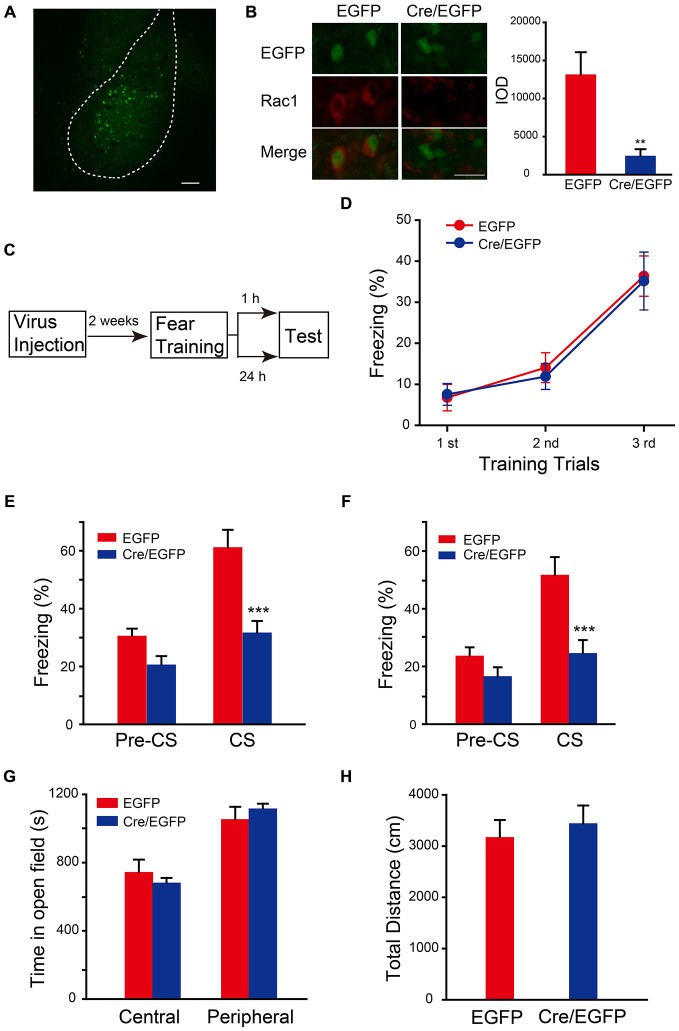
**Conditional knock out of Rac1 in BLA disrupted STM and LTM of fear conditioning. (A)** Fluorescence image of AAV infected BLA. Scale bar: 100 μm. **(B)** Immunofluorescence staining of Rac1 (red) in BLA of Rac1^flox/flox^ mice infected with AAV and the quantification of integrated optical density (IOD) of Rac1 in EGFP positive cells (*n* = 20 subjects each, **p < 0.05). Scale bar: 20 μm. **(C)** Experimental design. Two weeks after virus injection, mice were subjected to fear conditioning training followed by 1 h or 24 h memory test. **(D)** The freezing levels during each tone presentation in the training session (EGFP: *n* = 16, Cre/EGFP: *n* = 18). **(E,F)** Ablation of Rac1 in BLA decreased the freezing levels at 1 h (**E**, EGFP: *n* = 8, Cre/EGFP: *n* = 11, ***p < 0.001) and 24 h (**F**, EGFP: *n* = 8, Cre/EGFP: *n* = 9, ***p < 0.001) auditory fear memory tests. **(G,H)** The results of a 30 min locomotion test in an open-field apparatus (EGFP: *n* = 9, Cre/EGFP: *n* = 7).

The auditory fear memory is amygdala-dependent, and BLA contains a majority of spiny glutamatergic excitatory neurons (80%) and a minority of sparsely spiny GABAergic interneurons (20%; Orsini and Maren, 2012). Therefore, the potential role of Rac1 in BLA in STM and LTM formation was examined. AAV encoding Cre recombinase/EGFP was bilaterally infused into BLA of Rac1^flox/flox^ mice (Figure 2A). Expression of EGFP and Rac1 in BLA was detected 2 weeks after infection to verify the effect of viral expression. As shown in Figure 2B, Rac1 immunofluorescence in BLA significantly reduced in EGFP-positive neurons in Rac1^flox/flox^ mice infected with AAV-EF1α-EGFP-T2A-Cre (Figure 2B, *t*-test, *p* = 0.009). Two weeks after virus injection, mice were subjected to three-trial training for auditory fear conditioning followed by STM or LTM test (Figure 2C). During the training session, mice showed normal learning of fear memory (Figure 2D, two-way ANOVA, *F*_genotype×session(2,96)_ = 0.0555, *p* = 0.946). Rac1 conditional knock out in BLA of Rac1^flox/flox^ mice significantly decreased freezing percentage when presented with CS 1 h or 24 h after training, suggesting impaired STM and LTM (Figure 2E, 1 h, two-way ANOVA, *F*_genotype×session(1,34)_ = 5.993, *p* = 0.020; Figure 2F, 24 h, two-way ANOVA, *F*_genotype×session(1,30)_ = 5.416, *p* = 0.027; Bonferroni’s* post hoc* test). In the locomotion task, the conditional Rac1 knockout and control groups spent comparable time in the central and peripheral areas of the arena (Figure 2G, two-way ANOVA, *F*_(1,28)_ = 1.055, *p* = 0.313) and their total distance traveled in the open field has no difference (Figure 2H, *t*-test, *p* = 0.24), suggesting that amygdala-specific deletion of Rac1 does not change innate anxiety or motor activity. The above results indicate that Rac1 in BLA is required for both short-term and long-term fear memory formation.

### The Activation of Rac1 in BLA is Required for STM and LTM of Fear Conditioning

**Figure 3:**
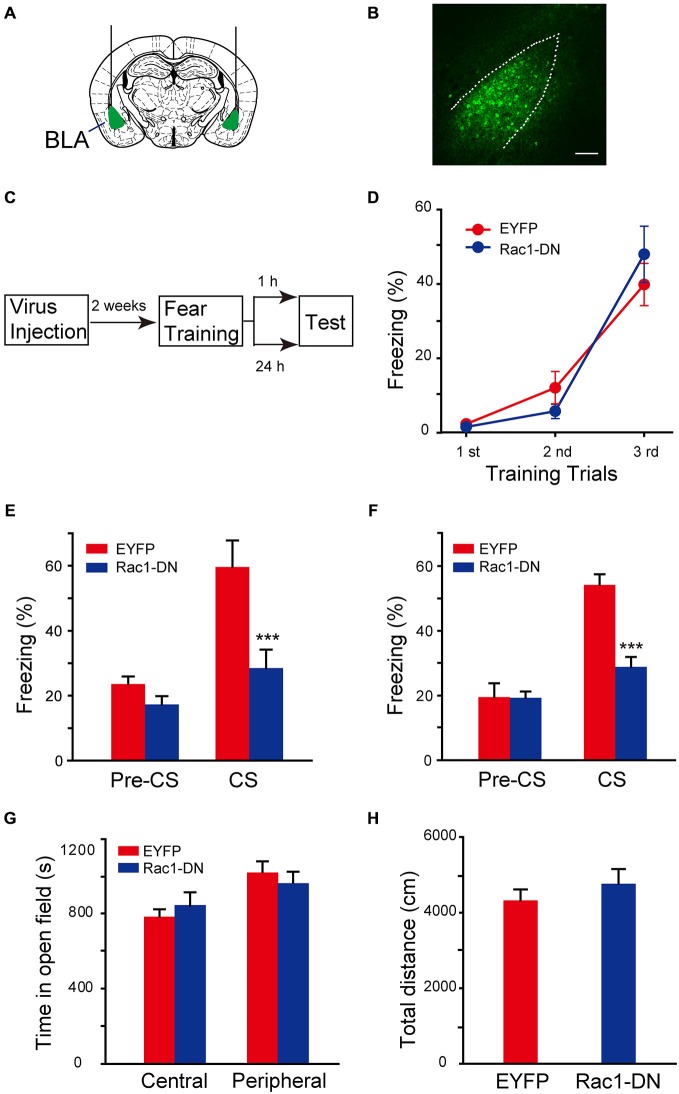
**Expression of Rac1 dominate-negative mutants in BLA blocked both STM and LTM of conditioned fear. (A)** Schematic representation of AAV injection sites. **(B)** Fluorenscence image of AAV infection in BLA. Scale bar: 100 μm. **(C)** Experimental design. Two weeks after AAV injection, mice were subjected to fear conditioning training followed by 1 h or 24 h memory test. **(D)** The freezing levels during each tone in training session (EGFP: *n* = 15, Rac1-DN: *n* = 14). **(E,F)** The expression of Rac1 DN mutant in BLA decreased the freezing levels at 1 h (**E**, *n* = 7 of each group, ***p < 0.001) and 24 h (**F**, EGFP: *n* = 8, Rac1-DN: *n* = 7, ***p < 0.001) auditory fear memory tests. **(G,H)** The results of a 30 min locomotion test in an open-field apparatus (EGFP: *n* = 11, Rac1-DN: *n* = 10).

The function of Rac1 GTPase in regulation of neuronal morphology depends on its activation. The requirement of activation of BLA Rac1 for fear memory formation was examined by approaches of expression of Rac1 dominant-negative mutant (Rac1-DN) and infusion of Rac1 inhibitor NSC23766 in BLA (Yao et al., 2011; Dietz et al., 2012). Rac1-DN, which competitively blocks GEF activation of Rac1, was cloned into AAV vector. The AAV encoding Rac1-DN/EGFP or control EGFP was bilaterally infused into BLA of C57BL/6 mice and the expression of EGFP reporter in BLA could be detected 2 weeks after viral infection (Figures 3A,B). Fear conditioning was conducted in these mice 2 weeks after the infusion of virus and the fear memory test was carried out 1 h or 24 h after fear conditioning (Figure 3C). In the fear conditioning training session, mice in both Rac1-DN and control EGFP infected groups displayed comparable fear learning during paired US-CS fear training and no significant difference between them was found (Figure 3D, two-way ANOVA, *F*_genotype×session(2,81)_ = 1.395, *p* = 0.254), indicating that disruption of Rac1 activation in BLA does not affect the initial learning of fear memory. However, when US-CS trained mice were re-exposed to CS 1 h or 24 h after training, the mice expressing Rac1-DN in the amygdala displayed significantly decreased freezing behavior compared with those infected with control virus (Figure 3E, 1 h, two-way ANOVA, *F*_genotype×session(1,24)_ = 5.521, *p* = 0.027; Figure 3F, 24 h, two-way ANOVA, *F*_genotype×session(1,26)_ = 14.122, *p* < 0.001). The open field test showed blockade of Rac1 activation did not affect anxiety or locomotion (Figure 3G, two-way ANOVA, *F*_(1,38)_ = 1.464, *p* = 0.234; Figure 3H, *t*-test, *p* = 0.386). These results suggest that the activation of Rac1 in BLA is required for both STM and LTM of conditioned fear.

**Figure 4:**
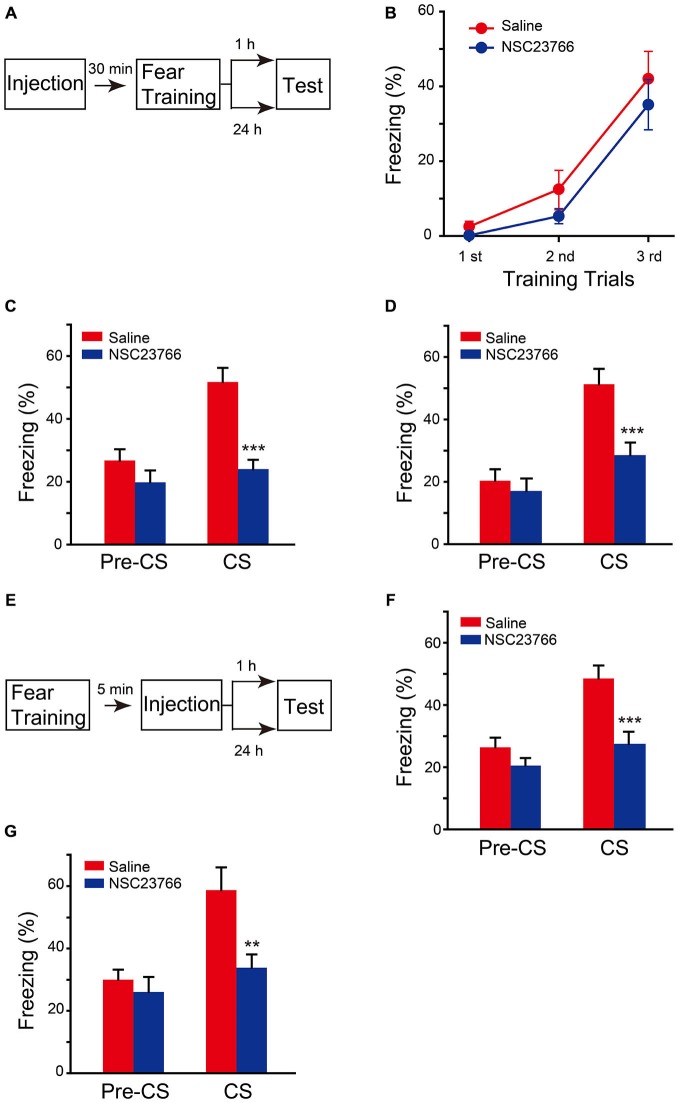
**Inhibition of Rac1 activity in BLA disrupted STM and LTM of fear conditioning. (A)** Experimental design for **(B–D)**. 30 min after NSC23766 injection, mice were subjected to fear conditioning training followed by 1 h or 24 h memory test. **(B)** The freezing levels during each tone in training session (Saline: *n* = 8, NSC23766: *n* = 11). **(C,D)** Inhibition of Rac1 activity in BLA decreased the freezing levels at 1 h (**C**, *n* = 7 of each group, ***p < 0.001) and 24 h (**D**, Saline: *n* = 10, NSC23766: *n* = 8, ***p < 0.001) auditory fear memory tests. **(E)** Experimental design for **(F,G)**. NSC23766 was injected 5 min after fear conditioning training and the memory test was performed 1 h or 24 h post training. **(F,G)** Post-training inhibition of Rac1 activity in BLA decreased the freezing levels at 1 h (Saline: *n* = 11, NSC23766: *n* = 10, ***p < 0.001) and 24 h (Saline: *n* = 6, NSC23766: *n* = 8, **p < 0.01) auditory fear memory test.

To confirm that the effect of chronic expression of AAV-Rac1-DN on fear memory is through direct suppression of Rac1 activation, but not through upregulation of Rac1-induced secondary effect, such as neuronal morphology changes in the brain, the effect of pharmacological blockade of Rac1 activation by NSC23766, an inhibitor of Rac1 activation by directly binding to Rac1 and prevent its activation by GEF, was determined. NSC23766 or saline was bilaterally infused into BLA 30 min before fear conditioning, and fear memory tests were performed 1 h or 24 h later after fear conditioning (Figure 4A). As shown in Figure 4B, NSC23766 pretreatment did not affect fear learning (Figure 4B, two-way ANOVA, *F*_genotype×session(2,54)_ = 0.345, *p* = 0.71), but decreased freezing when mice were presented with CS 1 h or 24 h after training (Figure 4C, 1 h, two-way ANOVA, *F*_drug effect×session(1,24)_ = 4.542, *p* = 0.044; Figure 4D, 24 h, two-way ANOVA, *F*_drug effect×session(1,32)_ = 5.202, *p* = 0.029). These results support the notion that activation of BLA Rac1 is required for STM and LTM formation.

Given that Rac1 inactivation before fear training impaired fear memory formation but did not affect the fear learning efficiency, we hypothesized that Rac1 activation in BLA after fear training may be critical for fear memory. Therefore, the effect of NSC23766 injected into BLA 5 min after fear conditioning was observed (Figure 4E). The NSC23766-treated group displayed significantly lower freezing than that of the saline group when presented with CS 1 h or 24 h after training (Figure 4F, two-way ANOVA, *F*_drug effect×session(1,38)_ = 4.728, *p* = 0.036; Figure 4G, two-way ANOVA, *F*_drug effect×session(1,24)_ = 4.345, *p* = 0.048), indicating that the activation of BLA Rac1 after fear learning is required for the formation of associative fear memory.

## Discussion

In the present study, we found that loss of Rac1 in excitatory neurons impaired STM and LTM of fear memory, and the ablation of Rac1 in BLA, a key brain region for auditory fear memory with abundant glutamatergic neurons, also impaired the formation of STM and LTM. Furthermore, the expression of Rac1 dominant-negative mutant or the injection of Rac1 inhibitor NSC23766 into BLA before fear conditioning blocked the formation of STM and LTM as well. In addition, infusion of NSC23766 into BLA after fear conditioning disrupted STM and LTM of fear memory. Our results showed that the post-training activation of Rac1 in BLA is critical for fear memory formation.

Studies have shown that Rac1 is involved in cocaine addiction. Repeated cocaine administration can negatively regulate Rac1 activity in the nucleus accumbens (NAc) and Rac1 signaling mediates structural and behavioral plasticity in response to cocaine exposure (Dietz et al., 2012). Injection of NSC23766 into the NAc core can inhibit the consolidation of cocaine-induced conditioned place preference (CPP) and injection of NSC23766 into BLA can disrupt the reconsolidation of cocaine-induced CPP (Ding et al., 2013). Other studies show the involvement of Rac1 in learning and memory. Intraperitoneal injection of the bacterial toxin cytotoxic necrotizing factor 1 (CNF1) to activate Rho GTPases, including Rac1, improves fear conditioning and spatial learning (Diana et al., 2007). Selective elimination of Rac1 in excitatory neurons in the forebrain impairs synaptic plasticity and Rac1 mutant mice display deficits in working/episodic-like memory in the delayed matching-to-place task (Haditsch et al., 2009). Injection of NSC23766 into BLA can disrupt the reconsolidation of auditory fear memory, while injection of NSC23766 into CA1 can disrupt the reconsolidation of contextual fear memory (Wu et al., 2014). But the role of Rac1 in fear memory learning and consolidation is not clear. Our study showed that the activation of BLA Rac1 is not required for fear memory learning, but necessary for both STM and LTM formation. However, Rac1 has been shown to be actively involved in forgetting and inhibition of Rac activity retards memory decay (Shuai et al., 2010). Our results do not exclude the possibility that the impairment of fear memories is due to disruption of memory retrieval or enhanced memory forgetting.

Rac1 can trigger activation of multiple signaling pathways (Bai et al., 2015). A classic downstream signaling of Rac1 is the PAK/LIMK pathway which regulates the dynamics of actin cytoskeleton (Nikolic, 2002). Activation of Rac1 can lead to polymerization of cerebral actin cytoskeleton. Accumulating evidence has shown that the actin cytoskeleton changes in fear memory formation in amygdala (Lamprecht, 2011). Infusion of actin cytoskeleton assembly inhibitors into the amygdala impaired the consolidation and reconsolidation of fear memory (Rehberg et al., 2010), and blocked the reacquisition when re-exposed to the US at the last extinction session (Motanis and Maroun, 2012). In addition, the synaptic plasticity is modulated by actin cytoskeleton, including the orchestration of the pre-synaptic vesicle cycle and the organization and trafficking of postsynaptic receptors (Kuriu et al., 2006; Xu and Wang, 2013; Gordon-Weeks and Fournier, 2014). Rac1 regulates dendritic spine morphogenesis, which is required for synaptic plasticity, through the modulation of actin cycling (Kennedy et al., 2005). These data suggest that Rac1 may mediate memory formation via modulation of actin cytoskeleton.

Our study shows that Rac1 is required for both STM and LTM formation, but inactivation of Rac1 before fear conditioning does not affect the apparent learning response during training trials, which is believed critical for memory acquisition. It has been proposed that the acquisition and STM formation processes are likely supported by distinct molecular mechanisms (Rodrigues et al., 2004). In addition, Rac1 is not only required for LTM but also STM formation, indicating that Rac1 is likely required by process in addition to morphology alterations for LTM formation (Lamprecht and LeDoux, 2004). In the hippocampus, the activation of Rac1 is increased 1 h after fear conditioning and remaining elevated 24 h after training (Diana et al., 2007), indicating the activation of Rac1 is long-lasting and persistent, which is critical for fear memory formation. Consistent with this proposal, our study found that the activation of Rac1, including post-training activation, is required for fear memory formation.

Taken together, our study revealed that Rac1 and its post-training activation in BLA are required for the formation of auditory fear memory. The downstream signaling pathways regulated by Rac1 in fear memory formation are remained to be elucidated. The study of the role of Rac1 in different memory processes may help to understand the development of memory in our brain.

## Funding

This research was supported by Ministry of Science and Technology Grants (2015CB553501, 2013CB835102, 2014CB942801), and National Natural Science Foundation of China Grants (91232307, 31571036, 31430033, 31421091, 31371136, 31270034).

## Conflict of Interest Statement

The Associate Editor Dr. Abumaria declares that, despite being affiliated to the same institution as the authors, the review process was handled objectively and no conflict of interest exists. The other authors declare that the research was conducted in the absence of any commercial or financial relationships that could be construed as a potential conflict of interest.
